# Dynasore, a Dynamin Inhibitor, Inhibits *Trypanosoma cruzi* Entry into Peritoneal Macrophages

**DOI:** 10.1371/journal.pone.0007764

**Published:** 2010-01-20

**Authors:** Emile S. Barrias, Lissa C. Reignault, Wanderley De Souza, Tecia M. U. Carvalho

**Affiliations:** Laboratório de Ultraestrutura Celular Hertha Meyer, Instituto de Biofísica Carlos Chagas Filho, Universidade Federal do Rio de Janeiro, CCS-Bloco G, Ilha do Fundão, Rio de Janeiro, Brasil; Fundação Oswaldo Cruz, Brazil

## Abstract

**Background:**

*Trypanosoma cruzi* is an intracellular parasite that, like some other intracellular pathogens, targets specific proteins of the host cell vesicular transport machinery, leading to a modulation of host cell processes that results in the generation of unique phagosomes. In mammalian cells, several molecules have been identified that selectively regulate the formation of endocytic transport vesicles and the fusion of such vesicles with appropriate acceptor membranes. Among these, the GTPase dynamin plays an important role in clathrin-mediated endocytosis, and it was recently found that dynamin can participate in a phagocytic process.

**Methodology/Principal Findings:**

We used a compound called dynasore that has the ability to block the GTPase activity of dynamin. Dynasore acts as a potent inhibitor of endocytic pathways by blocking coated vesicle formation within seconds of its addition. Here, we investigated whether dynamin is involved in the entry process of *T*. *cruzi* in phagocytic and non-phagocytic cells by using dynasore. In this aim, peritoneal macrophages and LLC-MK2 cells were treated with increasing concentrations of dynasore before interaction with trypomastigotes, amastigotes or epimastigotes. We observed that, in both cell lines, the parasite internalization was drastically diminished (by greater than 90% in LLC-MK2 cells and 70% in peritoneal macrophages) when we used 100 µM dynasore. The *T*. *cruzi* adhesion index, however, was unaffected in either cell line. Analyzing these interactions by scanning electron microscopy and comparing peritoneal macrophages to LLC-MK2 cells revealed differences in the stage at which cell entry was blocked. In LLC-MK2 cells, this blockade is observed earlier than it is in peritoneal macrophages. In LLC-MK2 cells, the parasites were only associated with cellular microvilli, whereas in peritoneal macrophages, trypomastigotes were not completely engulfed by a host cell plasma membrane.

**Conclusions/Significance:**

Taken together our results demonstrate that dynamin is an essential molecule necessary for cell invasion and specifically parasitophorous vacuole formation by host cells during interaction with *Trypanosoma cruzi*.

## Introduction

*Trypanosoma cruzi* is a flagellate protozoan that causes American trypanosomiasis, also known as Chagas' disease, which affects millions of people in Latin America. During its complex life cycle, the parasite has three morphologies (epimastigote, trypomastigote and amastigote forms) and alternates between invertebrate hosts (vectors) and vertebrate hosts such as mammals in which the infection is established [Reviewed in 1]. In vertebrate hosts, the trypomastigote is a highly infective form able to penetrate into all nucleated cells independently of their phagocytic capacity. Trypomastigote entry initially occurs through the formation of the parasitophorous vacuole. Interaction of this vacuole with endosomes and lysosomes takes place even during its initial formation, giving rise to a transient phagolysosome [Reviewed in 1]. The mechanisms by which *T. cruzi* is recognized and internalized, culminating in the formation of the phagolysosome, are still under debate. Accumulated evidence indicates that *T. cruzi* entry may occur by at least two basic processes: endocytosis/phagocytosis, in which the parasite is passively internalized through a classic endocytic pathway or by an active process in which the parasite is the agent of invasion. In both types of invasion, *T. cruzi* induces host cell PI 3-kinase (PI3K) activity [2], [3]. In addition, it has been shown that parasite entry may involve the participation of host cell membrane microdomains like flat domains (rich in flotillin proteins) and caveolae [4]. *T. cruzi* invasion also involves host cell assembly of actin microfilaments [5].

In mammalian cells, several molecules that selectively regulate the assembly of an endocytic vacuole have been identified. Among them, dynamin has been shown to play a major role in processes such as clathrin-mediated endocytosis [reviewed in 6], [7], synaptic vesicle recycling [8], phagocytosis [9], [10], transport from the trans-Golgi network [11] and ligand uptake through caveolae [reviewed in 12]. Dynamin is a GTPase family comprising three isoforms: dynamin 1, dynamin 2 and dynamin 3 [13]. All dynamins contain four domains: a GTPase domain (N-terminal), a pleckstrin homology domain (PH), a GTPase effector domain and a proline-arginine rich domain (PRD, C-terminal). The PH domain works as a binding motif for phosphatidylinositol 4,5–biphosphate, and the PRD domain mediates interaction with various proteins containing SH3 domains [14]. One protein class that interacts with dynamin is phosphatidylinositol 3-kinase (PI3K) [15]. Dynamin interacts with the p85 regulatory subunit of PI3K, and this interaction stimulates dynamin's GTPase activity. Gold and colleagues [9] reported that inhibition of PI3K prevents the recruitment of dynamin 2 to the site of particle binding, suggesting that, in phagocytosis, the activation of PI3K is upstream of dynamin. Among the three mammalian isoforms, dynamin 1 and dynamin 2 are the best characterized; however, despite extensive studies, the molecular mechanism by which dynamin participates in any of these processes is still a matter of debate [15]. According to some models, dynamin is a mechanochemical enzyme that is directly responsible for pinching off the vesicle [16]. According to others, it is a regulatory protein that recruits the downstream partner, which, in turn, drives the fission step [17]. Macia and colleagues [18], with the objective of identifying novel tools to study dynamin, discovered dynasore, a new reagent that has the ability to block the GTPase activity of dynamin. Dynasore noncompetitively inhibited the basal and stimulated rates of GTP hydrolysis without changing the GTP-binding affinity. Cells treated with dynasore showed a significantly decreased capacity to internalize transferrin and cholera toxin. The blockage is reversible and specific for dynamin-dependent endocytosis at the plasma membrane.

Wilkowsky and colleagues [17] showed, using dominant-negative dynamin (K44A) HeLa cells, that dynamin is involved in the invasion of *T. cruzi* in non-phagocytic host cells. However, since *T. cruzi* enters different host cells using a variety of different pathways, in view of the highly specific effect of dynasore, we decided to analyze its effect on the entry of *T. cruzi* into professional phagocytic and non-phagocytic cells. In view of the close functional connection between dynamin and PI 3-kinase activity, we also analyzed the effect of inhibitors of this enzyme on parasite attachment and penetration into macrophages. The effects of the various drugs on the morphology of the interaction process, as evaluated using high-resolution scanning electron microscopy and transmission electron microscopy, were also analyzed. The results obtained show that in both phagocytic and non-phagocytic cells, the process of *T. cruzi* entry into the host cell is drastically diminished when host cells are treated with dynasore, thus indicating the participation of dynamin in this process.

## Results

### Dynasore Blocks *Trypanosoma cruzi* Invasion in a Dose-Dependent Manner

**Figure 1:**
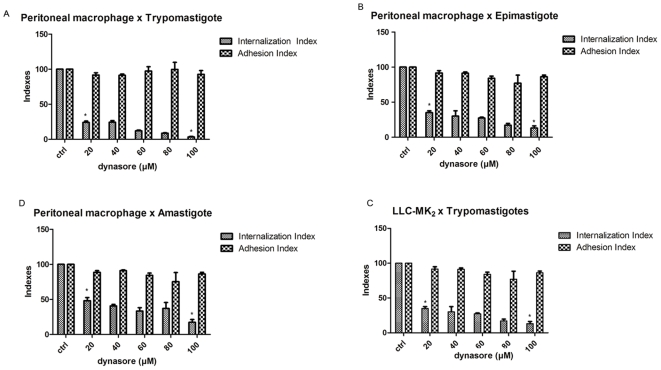
Dynasore impairs *Trypanosoma cruzi* internalization by host cells. Adhesion and internalization indexes of the interaction process betwen host cells treated for 20 minutes with increasing concentrations of dynasore (20, 40, 60, 80 and 100 µM) and exposed to *T. cruzi* (Y strain) A–C: After treatment with dynasore, peritoneal macrophages interacted with 10∶1 trypomastigotes (A), 10∶1 epimastigotes (B) or 10∶1 amastigotes (C) for 45 minutes, after which they were washed and stained with Giemsa. D: After treatment with dynasore, LLC-MK2 cells were allowed to interact with trypomastigote forms (10∶1). Quantification was carried out under a light microscope where 300 cells were counted in each coverslip, divided in two parameters: cells that present parasites inside and cells that present parasite attached to the cell surface. Each experiment was performed three times in duplicate. Values are the mean ± SD. Statistics were done by ANOVA and pair-wise comparisons were done by the Bonferroni test. p<0.05.

To determine whether dynamin is involved in *Trypanosoma cruzi* entry and in phagolysosome formation, cells were infected with the three *T. cruzi* forms (trypomastigotes, epimastigotes and amastigotes) in the presence of dynasore at varying concentrations. The peritoneal macrophages were treated with increasing amounts of dynasore or 0.005% DMSO (dymetilsulfoxide) and, after treatment, the medium containing the drugs was removed, and the parasites were added. Dynasore was removed before exposure to the parasites in order to guarantee that it affected only the host cell and not the parasites. After 15 minutes of incubation with parasites, which provides sufficient time for them to attach to cells, the free parasites were removed, and as dynasore activity is reversible after 20 minutes, the medium with increasing dynasore concentrations was added back until the end of the incubation period [18]. As shown in Figures 1 and S2, at all concentrations tested, dynasore did not interfere significantly with parasite adhesion but markedly inhibited the internalization of trypomastigote (A), epimastigote (B) and amastigote forms (C) by macrophages. Irrespective of concentration, the inhibition of internalization was more pronounced for the trypomastigote and epimastigote forms. For instance, at 80 nM, inhibition values of 98, 95 and 60% were observed for trypomastigote, epimastigote and amastigote forms, respectively. The same experiments were conducted using LLC-MK2 (non-phagocytic) cells and trypomastigotes. We observed that, even at 20 µM, dynasore reduced trypomastigote internalization by 55%, reaching 70% inhibition at 100 µM (Figure 1D). We observed no statistical differences in the adhesion and internalization indexes when we used pre-treated parasites with dynasore 60 and 100 µM before interaction with non treated host cells (Figure S1).

**Figure 2:**
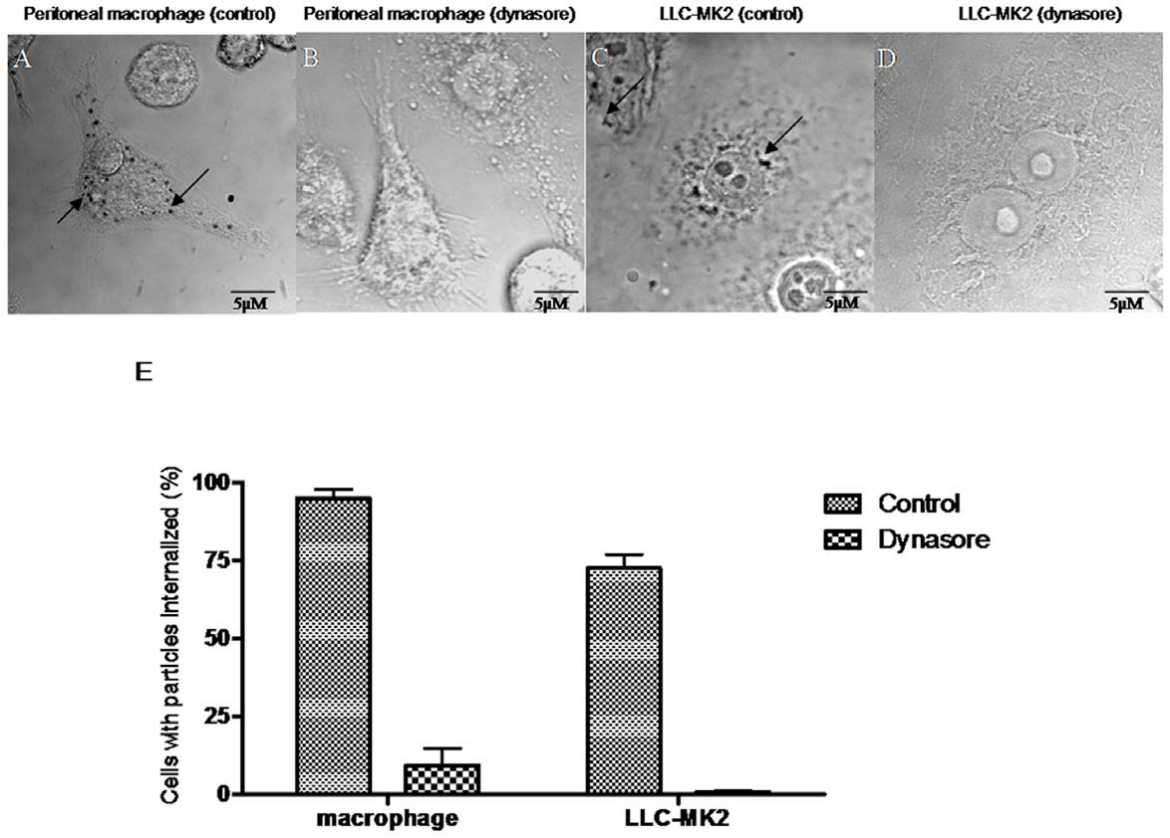
Dynasore inhibits endocytosis of BSA-Au by peritoneal macrophage and LLC-MK2 cell line. Observations by light microscopy of peritoneal macrophages and LLC-MK2 cells treated (or not) with 100 µM dynasore for 20 minutes and incubated with BSA-Au (10 nm, 5 µg/mL) for 45 minutes. A: Peritoneal macrophages without treatment. BSA-Au appeared inside the cells (arrows). B: Peritoneal macrophages treated with 100 µM dynasore. Inhibition of the GTPase dynamin by incubation with dynasore impaired BSA-Au entry. C: LLC-MK2 cells without treatment. BSA-Au appeared inside the cells (arrows). D: LLC-MK2 cells treated with dynasore. Inhibition of the GTPase dynamin prevented BSA-Au entry. E: Quantitative analysis of BSA-Au internalized by peritoneal macrophages or LLC-MK2 cells. The quantification was performed by light microscopy where 300 cells were counted in each coverslip. We divided in two parameters: cells that present BSA-Au molecules inside and cells that did not internalize BSA-Au. These experiments were done three times, each one in duplicate. The results were expressed in percentage of cells that present internalized particles or not. Values are the mean ± SD. Statistics were done by ANOVA and pair-wise comparisons were done by the Bonferroni test. p<0.05.

As a positive control, we also analyzed the effect of dynasore on the uptake of gold-labeled albumin by macrophages and LLC-MK2 cells, a process previously characterized as endocytosis (Figure 2A–D). As shown by light microscopy, while control cells (Figure 2A–C) showed a large number of ingested gold particles, very few or no particles were seen in dynasore-treated macrophages (Figure 2B), and no particles were seen in LLC-MK2 cells (Figure 2D). A quantitative analysis showed 80% inhibition with 100 nM dynasore in peritoneal macrophages and 100% inhibition in the LLC-MK2 cell line (Fig. 2E).

**Figure 3:**
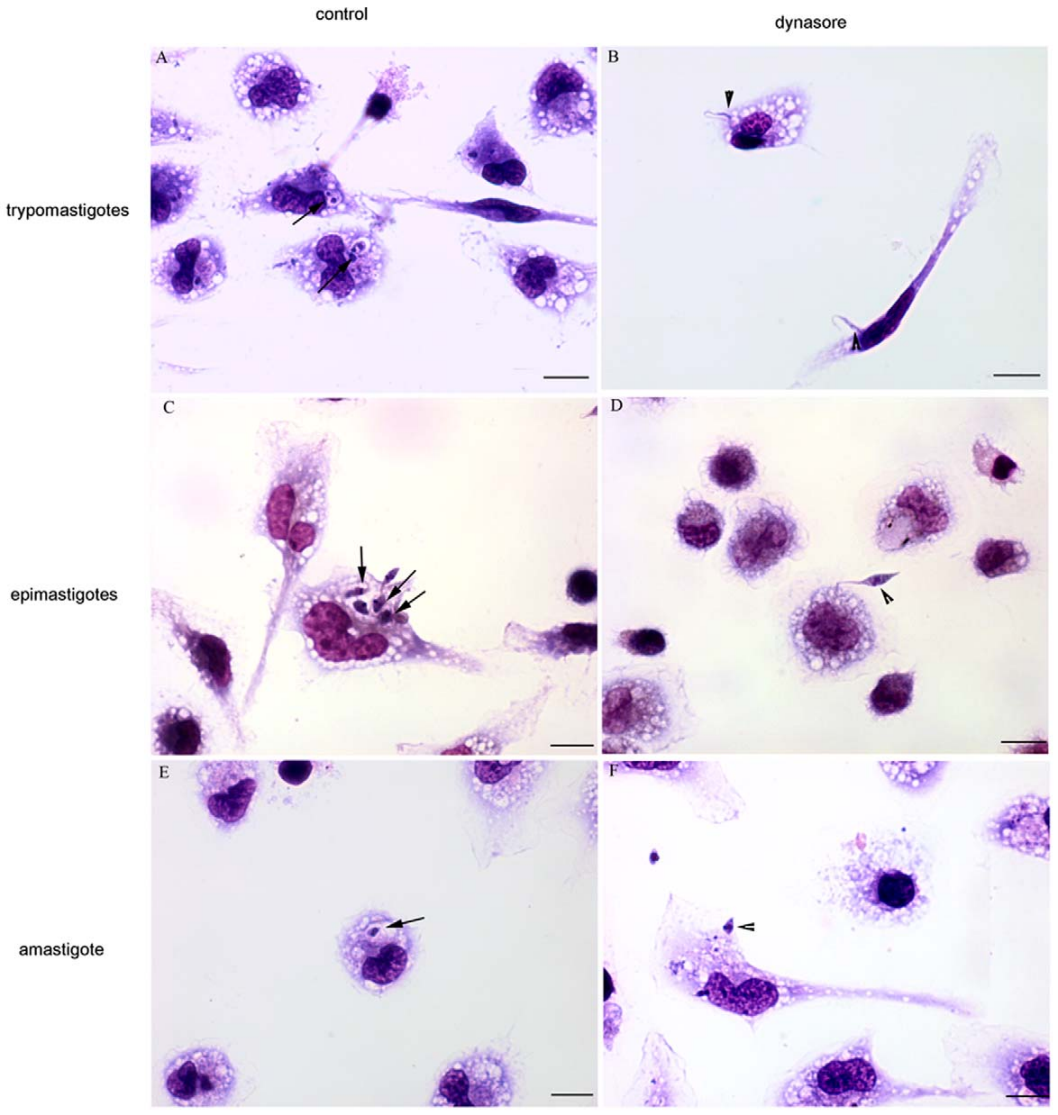
Light microscopy observations confirms *T cruzi* inhibition invasion of dynasore treated host cells. Observation after Giemsa staining by light microscopy of the interaction process between peritoneal macrophages treated (or not) with 60 µM dynasore for 20 minutes and exposed to different stages of *T. cruzi* (45 minutes). A, C and E: Peritoneal macrophages without treatment (control) and exposed to trypomastigotes (A), epimastigotes (C) and amastigotes (E). B, D and F: Peritoneal macrophages treated with 60 µM dynasore and exposed to trypomastigotes (B), epimastigotes (D) and amastigotes (F). The black arrows indicate internalized parasites, and the arrowheads indicate adhered parasites. Bars = 10 µm.

Light microscopy observation showed that, in dynasore-treated cells, all *T. cruzi* forms remained attached to the host cell surface (Figure 3). In some cases, we had the impression that part of the protozoan was in the process of being internalized by the macrophages.

### Dynasore Blocks the Formation of the Parasitophorous Vacuole

**Figure 4:**
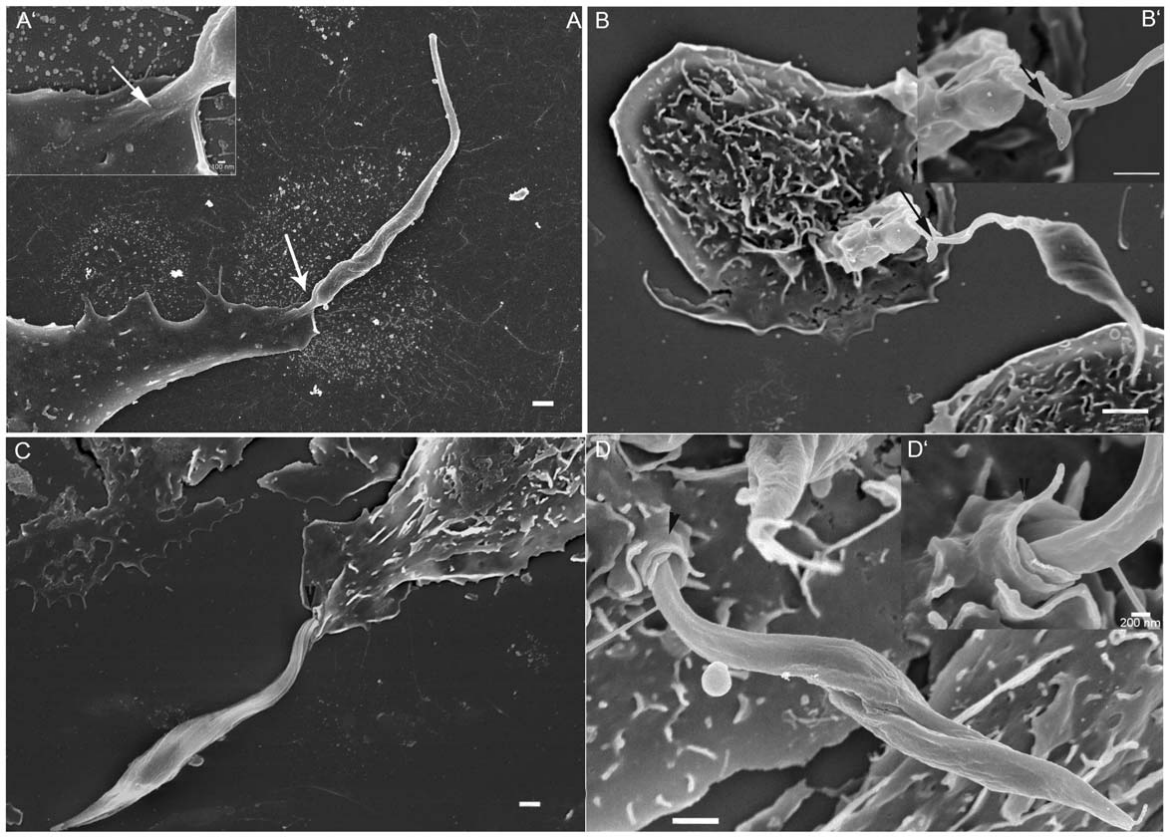
*Trypanosoma cruzi* can enters into host cells both by anterior and posterior ends. Observation by field emission electron microscopy (FESEM) of control peritoneal macrophages and trypomastigotes or epimastigotes. A: Trypomastigote invasion by the posterior body region (white arrow). B: Trypomastigote invasion by the anterior body region (white arrowhead). C and D: Epimastigote internalization by the flagella (anterior region - black arrow and arrowhead). Note that in D the epimastigote is internalized by coiled phagocytosis (black arrowhead) and in C the epimastigote is internalized by a funnel-like host cell plasma membrane structure (black arrow). Bars = 1 µm.

**Figure 5:**
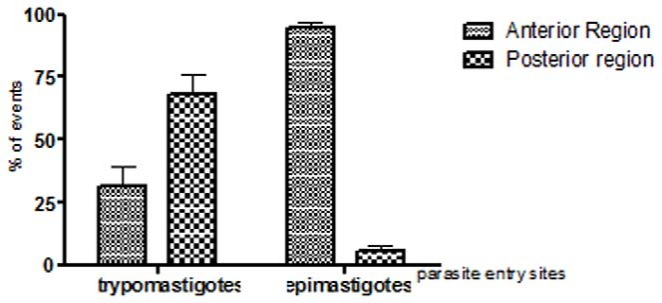
Quantitative analysis of trypomastigotes and epimastigotes entry's site in peritoneal macrophages. Trypomastigotes enter macrophages mainly by posterior region, while epimastigotes utilize preferentially anterior region. Values are the mean ± SD.

**Figure 6:**
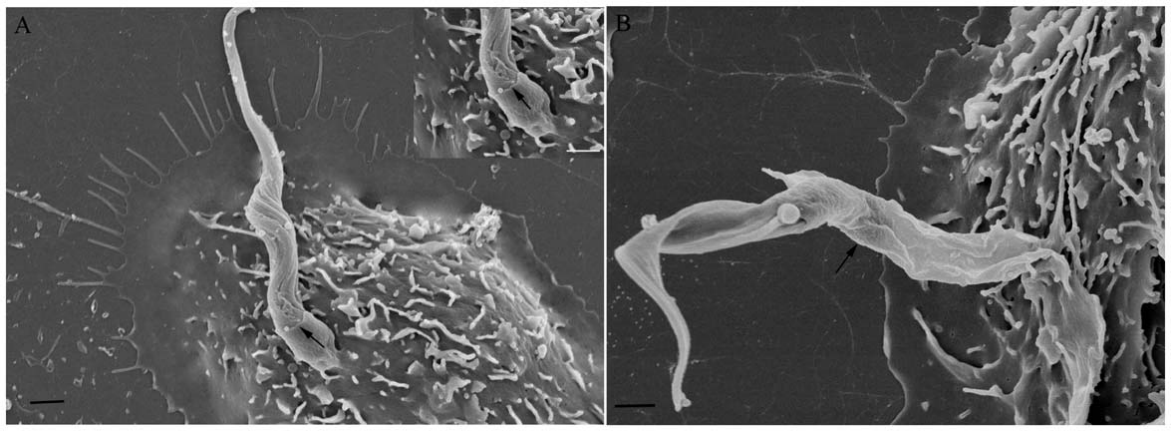
Field emission electron microscopy observations of the interaction process between peritoneal dynasore treated macrophages and *T. cruzi* trypomastigotes. After macrophage treatment with 60 µM of dynasore for 20 min, these cells were put to interact with trypomastigotes (120 minutes), washed and processed to FESEM. The parasites were covered by macrophage plasma membrane forming tubular structures around them (arrows). In A, the parasite is recovered by the macrophage plasma membrane observing a small portion of the parasite body recovered by the plasma membrane while in B we observed a large portion of partion of parasite, indicating that the bockage of GTPasic dynamin activity did not impairs the pseudopds extension impairing only the complet vacuole formation. The interaction time is enough to complete the parasite entry into control macrophages. Bars = 1 µm.

**Figure 7:**
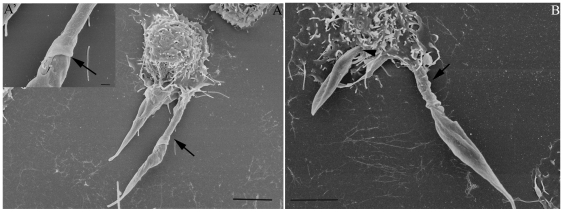
Field emission electron microscopy observations of the interaction process between peritoneal dynasore treated macrophages and *T. cruzi* epimastigotes. Field emission electron microscopy of the interaction between peritoneal macrophages treated with 60 µM of dynasore (for 20 minutes) and *T. cruzi* epimastigotes (Y strain). After macrophage treatment, these cells were allowed to interact with epimastigotes (120 minutes), before being washed and processed to FESEM. The parasites were covered by macrophage plasma membrane forming tubular structure around them (arrows). This tubular structure is linked tightly with the parasite. Note that epimastigotes can be internalized by treated macrophages without any host plasma membrane projection (B; arrowheads). The interaction time is enough to complete parasite entry. Bars = 1 µm.

**Figure 8:**
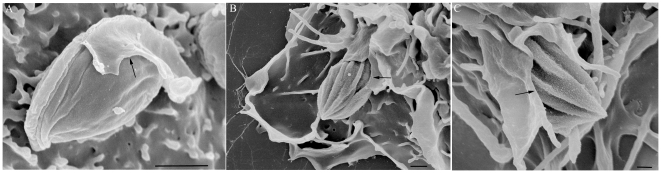
Field emission electron microscopy observations of the process interaction between peritoneal dynasore treated macrophages and *T. cruzi* amastigotes. Field emission electron microscopy of the interaction between peritoneal macrophages treated with 60 µM of dynasore (during 20 minutes) and *T.cruzi* amastigotes (Y strain). After macrophage treatment, these cells interacted with amastigotes (120 minutes), then were washed and processed to FESEM. In **A**, the parasite was enveloped by the macrophage plasma membrane (arrow). In B and C we observed a large portion of partion of parasite, indicating that the bockage of GTPasic dynamin activity did not impairs the pseudopds extension impairing only the vacuole closer (arrows). Bars = 1 µm.

**Figure 9:**
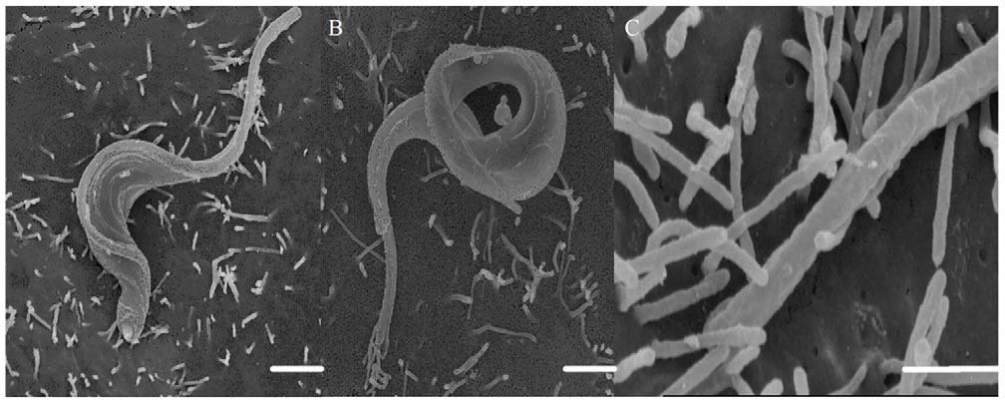
Dynasore treated LLC-MK2 shows only attached parasites. Field emission electron microscopy of the interaction between LLC-MK2 treated with 60 µM of dynasore (during 20 minutes) and *T .cruzi* trypomastigotes (Y strain). After LLC-MK2 treatment, these cells interacted with trypomastigotes (120 minutes), were then washed and processed to FESEM. The parasites are only adhered. Note that adhesion regions are involved by LLC-MK2 membrane projections. Bars = 1 µm.

Field emission scanning electron microscopy showed that even after a short interaction time, all developmental stages of *T. cruzi* are readily ingested by the macrophages. After 15 minutes of interaction, a variety of interaction types could be distinguished morphologically. In the case of trypomastigote forms, most of them entered the macrophages with their posterior region, where the kinetoplast is located, pointing towards the host cell (Figure 4A). However, some of them entered through the anterior flagellar region (Fig. 4B). Quantitative analysis showed that 65% of entry events occurred via the posterior region (Figure 5). Epimastigotes were internalized mainly via the flagellar region (Figure 4D). The macrophages' plasma membrane recovers the parasite by forming a funnel-like structure (Figure 4D) or with a structure previously described as a coiled-coil phagosome by Rittig and colleagues [19] (Figure 4C). Amastigotes did not show a preferential region of entrance. After 2 hours of interaction, no attached parasites were seen since the parasites had been ingested. In contrast, in dynasore-treated macrophages, very few parasites were internalized. However, a significant number of parasites attached to the macrophage surface and triggered the assembly process of filopodium-like, lamelopodium-like and even funnel-like structures. Images were obtained showing clearly that many parasites were partially internalized. In dynasore-treated macrophages, those few trypomastigote forms that were internalized preferentially used their posterior region (Figure 6A–B). Trypomastigotes were always partially covered by the macrophage plasma membrane. Similar experiments carried out with the non-infective epimastigote form showed the host cell plasma membrane covering mainly the flagellar regions (Figure 7A–B). In the case of amastigotes, internalization by dynasore-treated cells took place across the whole protozoan surface (Figure 8A–C). When the same experiments were conducted using the LLC-MK2 cell line, the parasites appeared only attached to the host cell surface, with host cell microvilli starting to engulf the parasite (Figure 9A–C).

**Figure 10:**
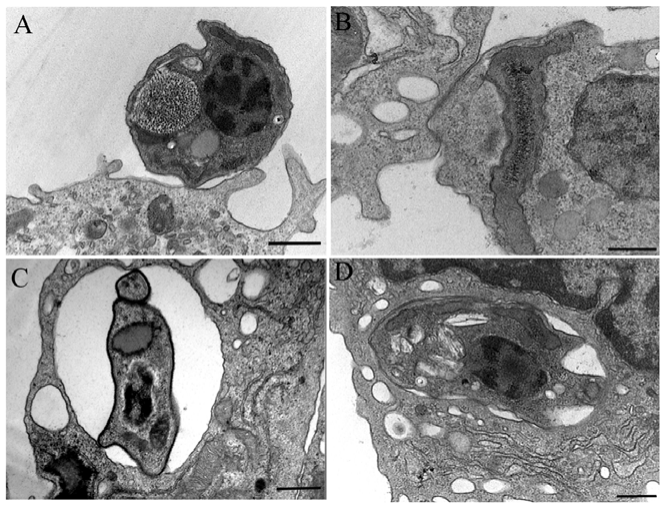
*T. cruzi* adhesion and internalization in dynasore treated macrophages. Transmission electron microscopy of peritoneal macrophages treated with dynasore 60 µM before interaction with trypomastigotes (A and C) or amastigotes (B). In A (trypomastigote) and B (amastigote), parasites were only attached at the macrophage plasma membrane's. C represents an internalized trypomastigote. Note that when compared with control (D) the vacuole observed in C is loose and appears in the periphery of macrophage. Bars = 0,5 µm.

**Figure 11:**
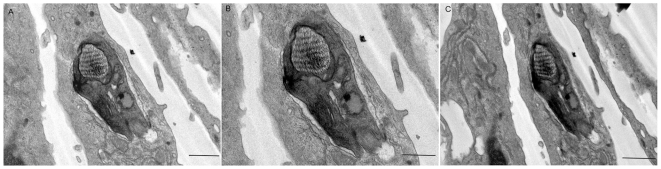
Vacuole containing trypomastigotes are completely closed. Electron micrographs taken at +4° (A), 0° (B) and −4° (C), using a goniometer, of macrophages treated with dynasore and infected with trypomastigotes of *T. cruzi*. Bars = 0,5 µm.

Transmission electron microscopy of thin sections showed the presence of trypomastigote (Figure 10A) and amastigote (Figure 10B) forms attached to the macrophage surface after two hours of incubation in the presence of dynasore. Those few internalized parasites seen in macrophages treated with dynasore were found in large vacuoles located at the cell periphery (Figure 10C), in contrast to the interaction with untreated macrophages (Figure 10D) where trypomastigotes appeared in the central portion of the cell. We considered whether these vacuoles were completely closed using goniometry and confirmed that this was in fact the case (Figure 11). In the case of attached parasites, close contact between the parasite and the macrophage membrane took place. Surface macrophage projections were also seen around the parasites.

### PI 3-Kinase Inhibitors Block the Formation of the Parasitophorous Vacuole in the Same Manner as Dynasore

**Figure 12:**
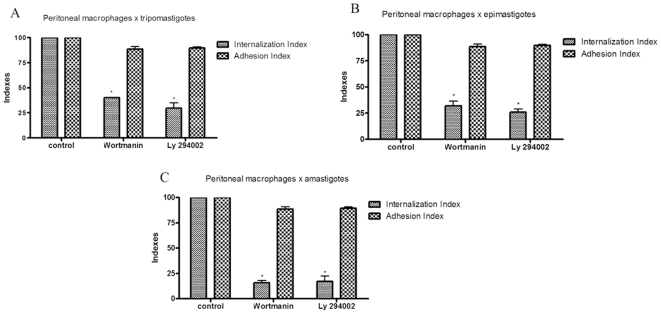
PI-3 Kinase inhibitors impair parasite internalization by macrophages. Adhesion and internalization indexes of the interaction process between peritoneal macrophage treated with wortmanin 10 nM or LY294002 100 µM (during 30 minutes) and *T. cruzi* (Y strain). After peritoneal macrophages treatment, these cells interacted with 10∶1 trypomastigotes (A), 10∶1 epimastigotes (B) or 10∶1 amastigotes (C) for 45 minutes and were then washed and stained with Giemsa. The quantification was carried out under light microscope where 300 cells were counted in each coverslip, divided in two parameters: cells that present parasites inside and cells that present parasite attached to the cell surface. Each experiment was performed three times in duplicate. Values are the mean ± SD. Statistics were done by ANOVA and pair-wise comparisons were done by the Bonferroni test. p<0.05.

**Figure 13:**
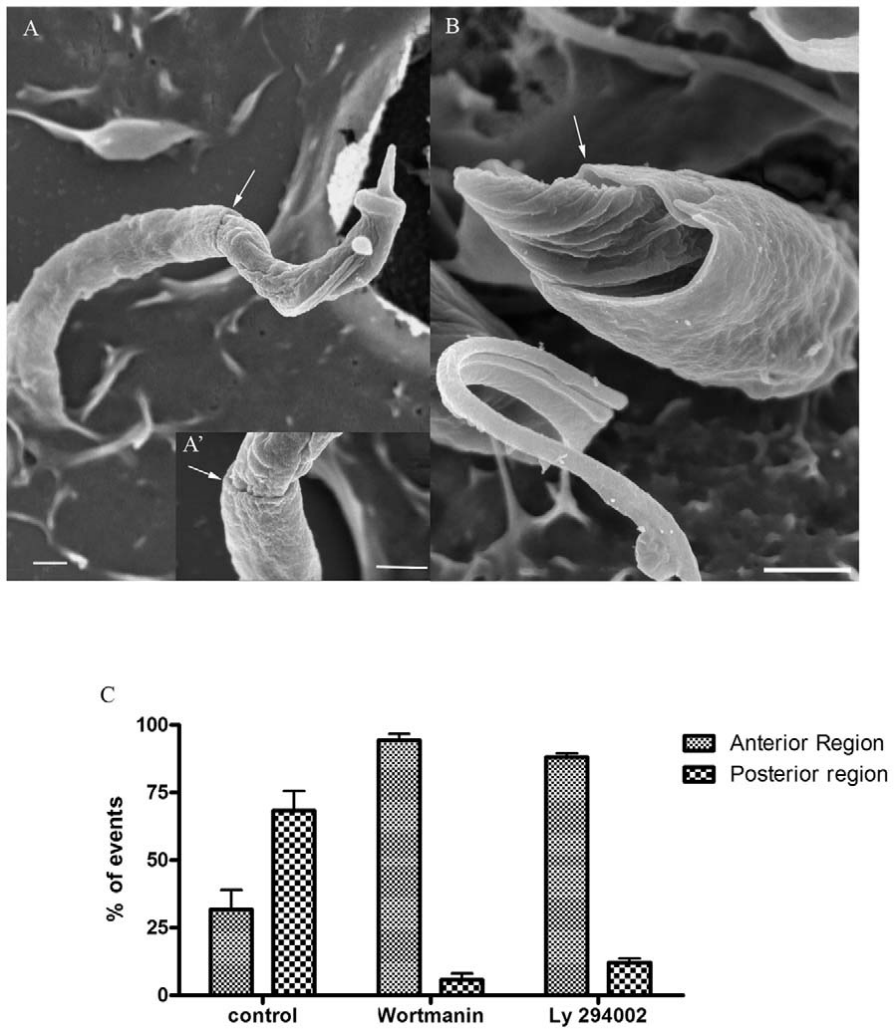
PI-3 Kinase inhibitors impair the vacuole complete formation and also change the parasite's entry preferential site. Field emission electron microscopy of peritoneal macrophage treated with wortmannin 10 nM interacting with trypomastigotes and epimastigotes. In A, A′ and B micrographs showing that trypomastigote and epimastigote appear recovered with a tight tubular structure formed by the macrophage membrane. Tubular structure was never observed sealing the entire parasite. (arrows). Bars = 1 µm. C: quantitative analysis of trypomastigotes body's region used to invade macrophages. Each experiment was performed three times in duplicate and a hundred interaction' events were quantified by FESEM. Values are the mean ± SD.

In view of the well-established close connection between dynamin and PI 3-kinase activation [9], we also analyzed the effect of wortmannin and LY294002, two well-characterized inhibitors of PI 3-kinase, on the *T. cruzi*-macrophage interaction process. Kinetic studies showed that both drugs inhibit parasite internalization by 60, 65 and 70% for epimastigote, amastigote and trypomastigote forms, respectively (Fig. 12). Scanning electron microscopy showed that trypomastigotes and epimastigotes remained attached to the macrophage surface, with plasma membrane extensions covering the parasites' bodies (Fig. 13A–B). In contrast to what happened with control macrophages in which trypomastigotes entered mainly through the posterior region, in drug-treated cells the trypomastigotes entered mainly through the anterior region. However, in the case of epimastigotes, PI3K inhibitors did not interfere with the entry pattern (Figure 13C).

## Discussion

One fundamental element in the life cycle of intracellular parasites, as is the case for *Trypanosoma cruzi*, is the mechanisms that they use to infect the host cells. The available evidence indicates that this process involves several steps, including (a) initial contact of the parasite to the cell surface, (b) attachment, (c) triggering of early host cell response that includes protein phosphorylation and assembly of surface cell projections, a process in which actin microfilaments are involved, (d) scission of the large endocytic vacuole containing the parasites and (e) interaction of endosomes/lysosomes from the host cell with the endocytic vacuole in formation. Previous studies have identified macromolecules exposed on the *T. cruzi* surface that are involved in the interaction process [reviewed in 20]. However, up to now, a host cell receptor has not been well characterized, although experimental evidence points to a role for laminin and fibronectin binding sites [21]. The involvement of different kinases [2] and the participation of actin filaments [5] in the interaction process have been well established. In addition, the interaction of organelles of the endocytic pathway with the parasitophorous vacuole in formation has been confirmed with the use of markers such as Rab5 and Rab7 [17]. HeLa cells (K44A) with increased GTP binding and hydrolysis showed a significant reduction in trypomastigote invasion [17].

Our present observations showing that previous treatment of macrophages with dynasore significantly inhibited internalization of all developmental stages of *T. cruzi* strongly support the idea that the host machinery involved in completion of the assembly of an endocytic vacuole plays a fundamental role in the process of parasite invasion. It has been shown that dynasore impairs the normal pinching off at the neck of the plasma membrane of the nascent parasitophorous vacuole, a process in which GTPases of the dynamin family play a key role by interfering both with initial vesicle formation and with vesicle liberation [18]. The effect was observed only when the host cells entered in contact with dynasore. One unexpected result is the fact that, although dynasore inhibited invasion, it did not cause an increase in the number of parasites attached to the macrophage surface.

Dynasore inhibition of *T. cruzi* penetration into macrophages was more evident in the infective trypomastigote forms, for which inhibition of up to 98% was observed at a concentration of 80–100 µM. This is the highest inhibition value reported up to now for interaction of *T. cruzi* with host cells. Studies using cytochalasin D [5], jasplakinolide [5], PI 3-kinase inhibitors [3], [22], negative dominant mutations of PKB, antibodies recognizing parasite molecules such as Tc85 [23] and cytokeratin 18 [24] reached inhibition values of 73%, 86% 40%, 88% and 30%, respectively. We considered whether dynasore might be affecting the parasite directly during when the medium was added back by incubating parasites with the drug. We found no evidence of dynasore adversely affecting the parasites themselves at the concentrations used (data not shownFigure S1????) Previous studies using dynasore have shown that dynamin is involved in the infection of mammalian cells by papillomavirus [25] and in phagocytosis by Sertoli cells [15]. In all cases, treatment of the host cells with dynasore significantly inhibited internalization.

Our morphological observations, using high-resolution scanning electron microscopy of macrophages allowed to interact with *T. cruzi*, showed significant variation in the pattern of interactions of the various developmental stages. While the trypomastigote form is preferentially internalized by peritoneal macrophages using its posterior region, epimastigotes are internalized via the flagella. This difference was shown here for the first time using a quantitative approach. It is possible that it is due to different mechanisms of ingestion of the two developmental stages of *T. cruzi* by the macrophages.

Previous incubation of the macrophages with dynasore did not change the pattern of interaction of the parasites with the macrophages.

The analysis of the process of pinching off of vesicles formed during the formation of endocytic vesicles and vacuoles has shown that PI3K is involved in the whole process [9]. Using drugs that inhibit PI3K, such as wortmannin and LY294002, it has been suggested that activation of PI3K is upstream of dynamin [9]; inhibition of PI3K inhibited the complete sealing of surface projections that participate in the endocytic process. It has also been shown that the close association between PI(4,5)P2-bound dynamin 2 and actin dynamics modulation results in the assembly of lamellipodia and ruffles [15]. Previous studies have shown that treatment of macrophages with drugs that inhibit PI3K activity also inhibited *T. cruzi* internalization. Our present observations also confirm these results.

We also observed that the few parasites that entered into dynasore-treated cells remained at the cell periphery and did not move to the more central portion of the host cell, where the nucleus is located, as occurs in untreated cells. We do not have a clear explanation for this fact, but it is possible that inhibition of the dynamin system also interferes in some way with the host cell cytoskeleton that participates in the traffic of the initial parasitophorous vacuole from the cell periphery to its most central portion. Dynasore inhibition could be avoided by washing the cells before host cell infection, which is in agreement with the recovery of transferrin endocytosis observed by Macia *et al*. [18].

Two observations made by transmission electron microscopy of dynasore-treated macrophages allowed to interact with *T. cruzi* deserve comment. First, actin polymerization took place immediately below the macrophage membrane at sites of contact with the parasites, a result that is in close agreement with previous results reporting actin redistribution (shown by immunofluoresccence) and the effect of previous treatment of the cells with cytochalasin D before interaction [5]. Second, the few trypomastigotes able to penetrate into dynasore-treated cells remained in large peripheral vacuoles.

In conclusion, the use of dynasore allowed us to show clearly that the host cell plays an active and important role in the process of *Trypanosoma cruzi* invasion.

## Materials and Methods

The experimental protocol was approved by the Instituto de Biofisica Carlos Chagas Filho (Universidade Federal do Rio de Janeiro) Ethics Committee for animal experimentation.

### Chemicals

Dynasore was kindly supplied by Dr. Tomas Kirchhausen (Department of Cell Biology, Harvard University, Boston, and IDI Research Institute, Boston, Massachusetts, USA). It was solubilized in DMSO to obtain a stock solution at 200 mM. Aliquots were stored at −20°C and diluted to final concentrations in the culture medium just before use. Wortmannin and LY294002 were purchased from the Sigma Chemical Company (St. Louis, MO, USA).

### Bovine Serum Albumin-Coloidal Gold Preparation

Coloidal gold particles (8–10 nm) were prepared as previously described [26]. Bovine serum albumin (BSA) (electrophoretic grade, Sigma Chemical Company, St Louis, MO, USA) were coupled to gold particles as described [27] and used at final concentration of 25 µg/mL in RPMI 1640 culture medium.

### Parasites and Cell Culture

*T. cruzi* trypomastigotes (Y strain) were derived from the supernatants of infected LLC-MK_2_ culture cells (ATCC CCL-7; American Type Culture Collection, Rockville, MD) grown in RPMI 1640 medium with garamycin (GIBCO, Grand Island, NY) and 10% fetal bovine serum (FBS), at 37°C in 5% CO_2_. Subconfluent cultures of LLC-MK2 cells were infected with 5×10^6^ trypomastigotes. Free parasites were removed after 24 h and the cultures were maintained in 10% FBS-RPMI 1640. Five days following infection, free trypomastigote forms could be found in the cell supernatants. After ten days, amastigote forms were observed in the cell supernatants. Epimastigote forms (Y strain) were cultivated in LIT medium as previously described [28], and after four days of cultivation, they were collected by centrifugation at 350 g.

Resident peritoneal macrophages were obtained from Swiss mice. They were collected using Hank's solution, plated on 13 mm round glass coverslips and allowed to adhere for 45 minutes at 37°C in a 5% CO_2_ atmosphere. Subsequently, non-adhered cells were removed by washing with Hank's solution, and RPMI 1640 medium with 10% FBS was added. The cells were maintained in culture for 24 hours at 37°C in 5% CO_2_ before experiments. In experiments using LLC-MK2 cells, the cells were plated on 13 mm round glass coverslips and washed with RPMI 1640 before the interaction assays.

### Dynasore Treatment

Before the experiments, peritoneal macrophages and LLCMK_2_ were washed three times with RPMI 1640 without serum and incubated for 20 minutes at 37°C in a 5% CO_2_ atmosphere in the presence of different concentrations (20, 40, 60, 80 and 100 µM) to test its effect on parasite adhesion and internalization into the host cells. To perform experiments involving microscopic analysis (light, scanning and transmission electron microscopy), dynasore was used at a 60 µM concentration. After 20 minutes, the medium containing dynasore was removed, and peritoneal macrophages or LLC-MK2 were allowed to interact with trypomastigote, amastigote or epimastigote forms added to achieve a ratio of 10 parasites per mammalian cell. The interaction lasted 15 minutes, at 4°C; the free parasites were then removed, and RPMI 1640 medium with varying concentrations of dynasore was added and left to incubate for 30 more minutes at 37°C in a 5% CO_2_ atmosphere. After the 45 minutes of interaction, the cells were washed three times and fixed for subsequent light or electron microscopy observation. All experiments included untreated infected peritoneal macrophages as controls. Similar experiments were carried out using gold-labeled BSA (25 µg/mL) in order to verify whether or not dynasore interferes with the endocytic uptake of the protein. Experiments were performed in duplicate, and three independent experiments were completed. The viability of the cells obtained from the cultures before and after incubation experiments was performed using Tripan blue assay (0.2% of trypan blue for 5 minutes). The quantification was carried out using light microscopy where a total of 100 cells were randomically counted.

### Wortmannin and LY294002 Treatment

Peritoneal macrophages were treated with 10 nM wortmannin or 100 µM LY294002 for 30 minutes before interaction and were then washed three times with RPMI 1640 medium without serum. The macrophages were then allowed to interact with *T. cruzi* trypomastigotes for 15 minutes (40°C), washed and allowed to interact for 90 more minutes (37°C/5%CO_2_). After this time, the macrophages were fixed and prepared for light or scanning electron microscopy. To quantify the body regions of *T. cruzi* used to attach to peritoneal macrophages, one hundred scanning electron micrographs obtained from experiments performed in triplicate were analyzed.

### Light Microscopy

For light microscopy, the cells were fixed with Bouin's fixative and stained with Giemsa (Merck). The cells were observed by bright field microscopy in order to distinguish attached from internalized parasites. The percentage of cells with attached and with internalized parasites and the mean number of parasites per cell were determined by randomly counting at least 600 cells in three independent experiments. The adhesion index was calculated by multiplying the percentage of cells with attached parasites by the mean number of attached parasites per cell. The endocytic index was calculated by multiplying the percentage of infected cells by the mean number of parasites per infected cell. All endocytic index were normalized.

To gold-BSA quantification was done by light microscopy where 600 cells were conted in each coverslip. We considered two parameters: cells that present BSA-Au particles inside and cells that did not present BSA-Au in its interior. These experiments were done three times, each one in duplicate. The results were expressed in percentage of cells that present or not particles in its interior.

### Electron Microscopy

For field-emission scanning electron microscopy, the host cells (peritoneal macrophages and LLC-MK2) were cultivated on 13 mm coverslips in 24-well plates. After interaction (15 minutes for cells without any treatment and 2 hours for cells treated with dinasore or wortmannin), the cells were washed and then fixed in a solution containing 2.5% grade I glutaraldehyde (TedPella) in 0.1 M cacodylate buffer, pH 7.2, for 30 minutes to 1 hour, post-fixed with 1% OsO_4_ in 0.1 M cacodylate buffer, pH 7.2, plus 0.8% potassium ferrocyanide (1 hour) dehydrated in ethanol series (50, 70, 90 and 100%), critical point-dried in a Baltec CPD 030 apparatus and mounted on specimen stubs. The samples were ion-sputtered to avoid charge effect with 2–3 nm gold layer and observed with a Jeol 6340 field emission scanning electron microscope operating at 5.0 kV and 12 µA.

For transmission electron microscopy, the cells were allowed to grow in 60 mm^2^ Petri dishes. After the experimental procedure, cells were fixed as described for scanning electron microscopy, dehydrated in increasing concentrations of acetone and flat-embedded in Polybed (Polysciences®). *En face* sectioning avoided removal of the cells from the substratum, which could disrupt and disorient their architecture. Ultrathin sections were stained with uranyl acetate and lead citrate and observed under a Zeiss 900 transmission electron microscope.

### Statistical Analysis

The statistical analysis was conducted using ANOVA with the Bonferroni test. Values are presented as mean ± SD. The results were considered significant when P<0.05.

## Supporting Information

Figure S1 (5.83 MB TIF)

Figure S2 (6.36 MB TIF)
