# Supplementary figures and images for: Dynasore, a Dynamin Inhibitor, Inhibits Trypanosoma cruzi Entry into Peritoneal Macrophages
Source: PLoS One. 2010 Jan 20;5(1):e7764. doi: 10.1371/journal.pone.0007764 (PMC2808331; doi:10.1371/journal.pone.0007764)

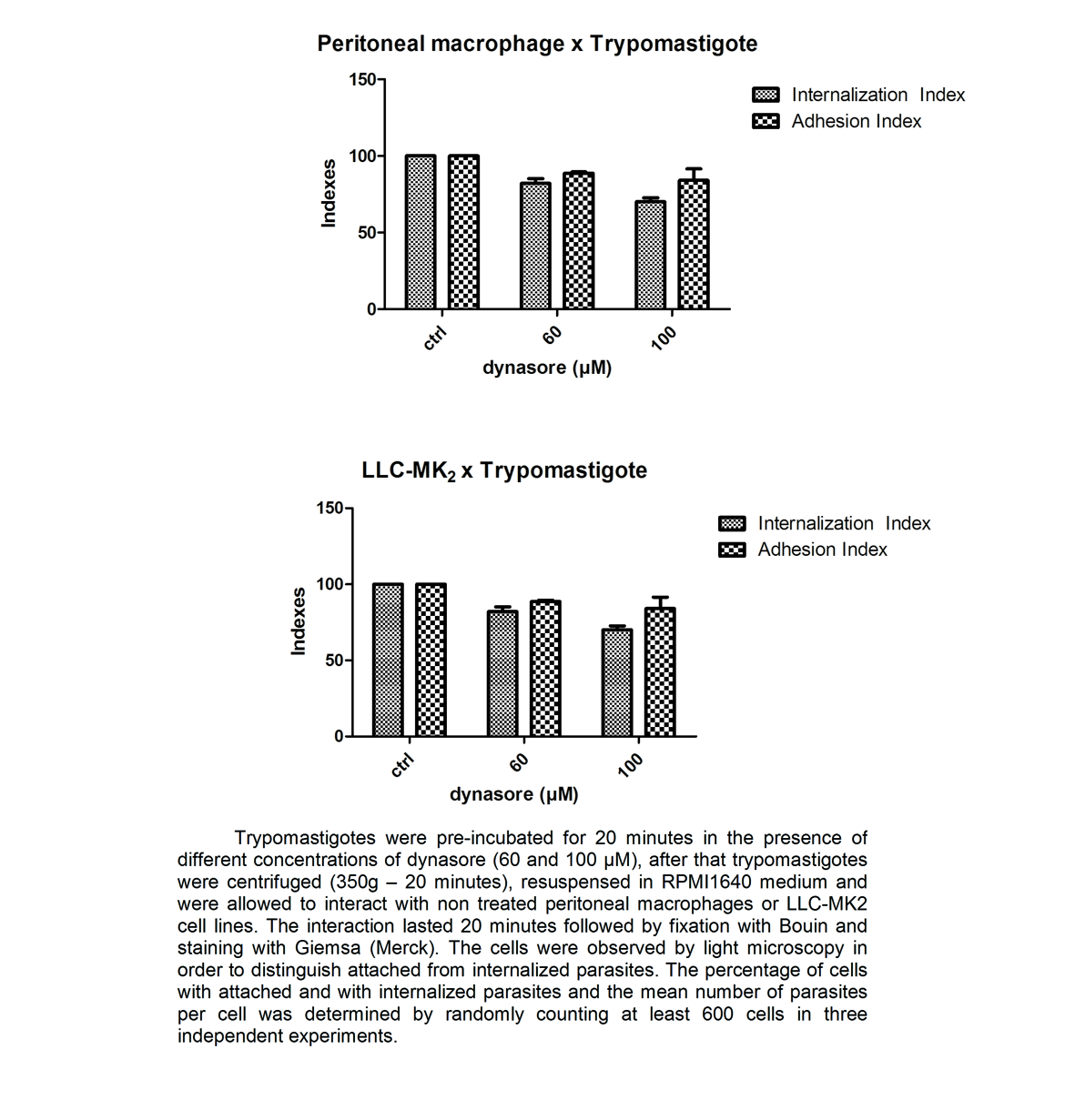

Supplement: Figure S1 — (5.83 MB TIF) [file pone.0007764.s001.tif]

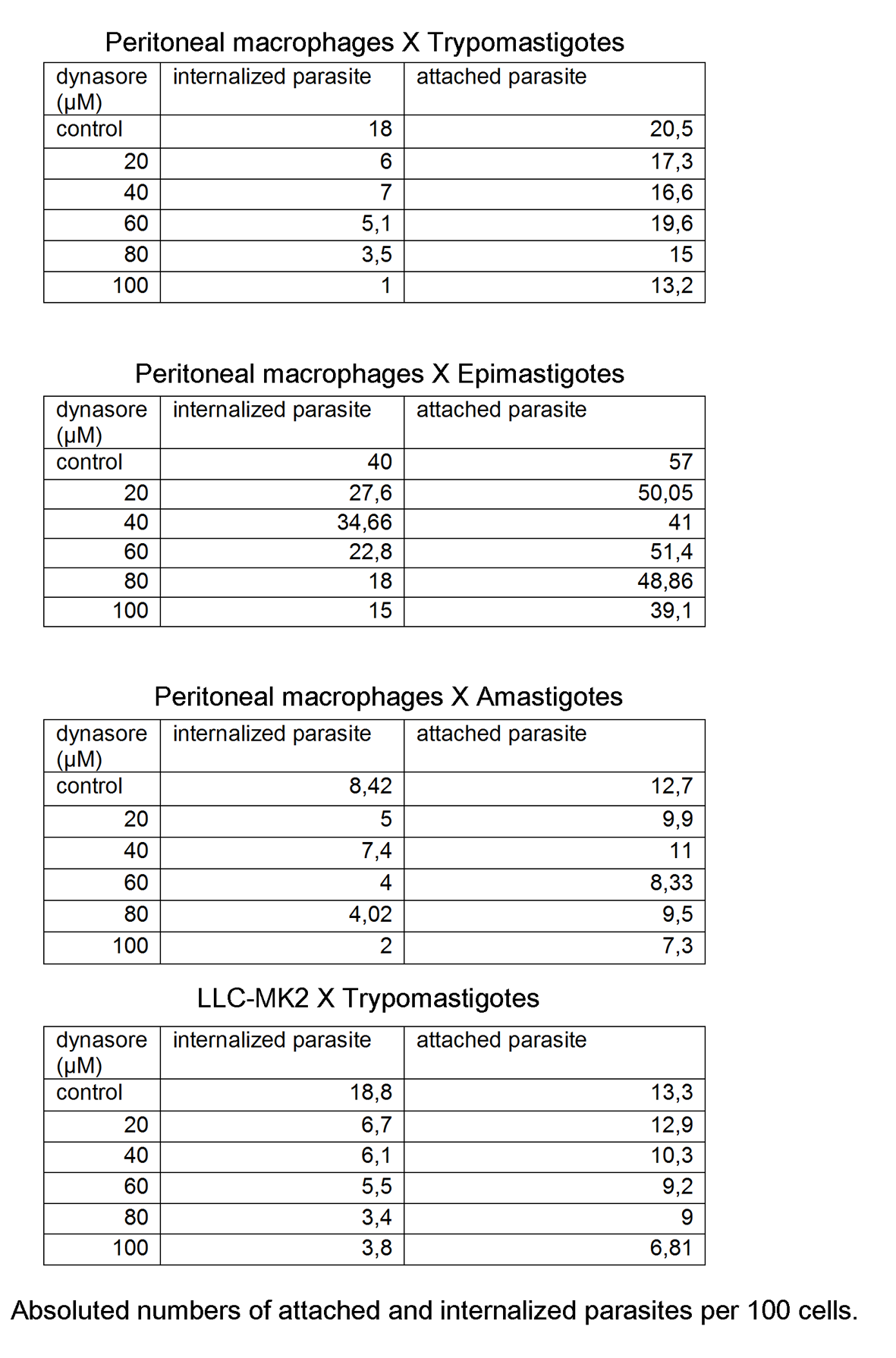

Supplement: Figure S2 — (6.36 MB TIF) [file pone.0007764.s002.tif]
